# Implications of a change in case definition and screening of asylum seekers for hepatitis B surveillance in Germany in 2015 and 2016

**DOI:** 10.1017/S0950268820000242

**Published:** 2020-02-24

**Authors:** A. von Laer, M. Diercke, M. an der Heiden, D. Altmann, R. Zimmermann, S. Dudareva

**Affiliations:** 1 Postgraduate Training for Applied Epidemiology, Robert Koch Institute, Berlin, Germany; 2 Department of Infectious Disease Epidemiology, Robert Koch Institute, Berlin, Germany; 3 European Programme for Intervention Epidemiology Training, European Centre for Disease Prevention and Control (ECDC), Stockholm, Sweden

**Keywords:** Epidemiological surveillance, Germany, hepatitis B, refugees

## Abstract

Since 2015, the number of hepatitis B virus (HBV) cases increased substantially in Germany. In 2015, a more sensitive HBV case definition was introduced. This coincided with an asylum seeker influx with differing screening strategies. Information on the asylum seeker status has been collected since 09/2015. We investigated this increase to interpret HBV notification data in Germany. We compared HBV surveillance data from 2010–2013 (baseline) with 2015–2016, excluding 2014 due to beginning of asylum seeker influx. We estimated the excess above the mean case number (baseline) using Poisson regression and compared asylum seeker cases and the excess of cases with the unknown asylum seeker status. HBV cases increased from 1855 (mean baseline) to 3873 (2015) and 3466 (2016) with 1903 asylum seeker cases and 1099 excess-cases with the unknown asylum seeker status in 2015–2016. Cases only fulfilling the changed case definition increased from 60% (1119) in baseline to 81% (*P* < 0.01) in 2015–2016; 69% of asylum seeker cases and 61% of excess-cases were males <40 years compared to 27% (baseline) (*P* < 0.01). Changed case definition increased the number of cases in official statistics substantially. Demographic and geographical distributions suggest that screening of asylum seekers increased the case numbers even to a higher extent than surveillance data indicates.

## Introduction

Viral hepatitis is a major public health challenge. According to the World Health Organization (WHO) an estimated 257 million people live worldwide with chronic hepatitis B [1]. Prevalence of active hepatitis B (hepatitis B surface antigen (HBsAg) positive) varies and is highest in Sub-Saharan Africa and South Asia with 5–10% [2]. In Europe, the prevalence of active hepatitis B is estimated to be 0.9%, corresponding to almost 4.7 million individuals living with hepatitis B [3]. The incidence of active hepatitis B in European countries varies between 0.1 and 23 per 100 000 inhabitants [4]. In Germany, vaccination against hepatitis B is recommended for all children above the age of 2 months since 1995. In 2017, 86.9% of all children were vaccinated as assessed during the school entry health examination [5]. Germany is a low prevalence country for hepatitis B and the last population-based survey in the adult general population in Germany (DEGS1, 2008–2010) found a prevalence of active hepatitis B of 0.3% [6].

Hepatitis B is a notifiable disease in Germany according to the Protection against Infection Act (IfSG). According to the IfSG, hepatitis B cases are mandatorily to be notified from the laboratory and the clinician to the LPHA irrespective of nationality or residence of the case. In the period under investigation only acute hepatitis B infections were notifiable to LPHA according to the IfSG.

In 2015, the case definition was changed to be more sensitive and to better comply with the ECDC case definition [7]. According to the case definition before 2015 the presence of clinical symptoms was necessary to constitute a case of hepatitis B, whereas according to the changed case definition since 2015 cases have to fulfil laboratory criteria only.

As it could be difficult to discrimante acute and chronic infection for laboratories, we believe that newly diagnosed hepatitis B virus (HBV) infections, irrespective of the infection stage, were notified from the laboratory to the LPHA even before 2015. The case definition is addressed to the LPHA and not to the laboratories. At LPHA cases are classified according to the case definition, further investigations are conducted and prevention measures are implemented [8]. Applying the case definition is supported by the notification software, but could be decided manually. To apply the changed case definition, a software update was necessary in each LPHA. The LPHA enters the data into the notification software including all important information, e.g. the asylum seeker status or the most probable country of infection. All cases fulfilling the laboratory criteria, irrespective of the clinical criteria, are transmitted electronically from the LPHA to the state public health authorities and from there further to the national public health institute (Robert Koch Institute, RKI) [9]. Only cases fulfilling the case definition are published nationally to inform stakeholders. According to the case definition before 2015 only cases fulfilling also the clinical criteria were published in the statistics, whereas according to the changed case definition since 2015 all cases fulfilling the laboratory criteria are published. Cases with an unknown infection stage (i.e. acute or chronic) fulfil the case definition and are included in published data, but cases that are known to be chronically infected in the notification data are excluded from publication.

In 2015, Germany experienced a large influx of asylum seekers. It is estimated that 890 000 asylum seekers came to Germany in 2015, mostly from Syria (36%), Albania (12%) and Kosovo (8%) [10, 11]. In these countries, the prevalence of active hepatitis B is higher than in Germany (5.6%, 9.5% and 2.4%, respectively) [12–14]. Most asylum seekers were young males: 69% were male, 90% were below 40 years [11, 15, 16]. In 2015, three federal states had included obligatory hepatitis B screening as part of their initial examination of asylum seekers [17–19]: Hamburg, Bavaria and Saxony. Thuringia recommended a hepatitis B screening only if indicated. Screening is implemented on a local level. Data on screening implementation is not available at the national level. Therefore, it is not known, to what extent these regulations have been implemented. In Bavaria, threefold more asylum seekers were screened for hepatitis B in 2015 compared to 2014 [18].

Since 2015, a large increase of published hepatitis B cases has been observed in Germany. We investigated to what extent this increase is attributable to the changed case definition in 2015 or the screening of asylum seekers to be able to better interpret notification data on hepatitis B in Germany.

## Methods

### Data and descriptive analyses

#### Data sources

We extracted and analysed all hepatitis B cases that were transmitted to RKI irrespective of the case definition between 2010 and 2016 from the national surveillance database Surv*N*et with data status as of 1 March 2017 [9].

Data on registered asylum seekers and refugees were obtained from publications from the German Federal Office for Migration and Refugees and the German Federal Agency for Civic Education [11, 15, 16, 20].

#### Data analysis

As a first step, we performed a descriptive analysis by time-period and federal state. Data from 2014 was excluded from the analyses, because the influx of asylum seekers started during 2014, but information on the asylum seeker status was not yet available in our notification data and the old case definition was still in place. We analysed the data using Stata^®^ version 14.1 and Microsoft Excel 2010.

#### Definitions

Each notification that has been transmitted to the RKI irrespective of the applied case definition was defined as a case. Cases that were transmitted as known chronic diseases were excluded from the analysis.

Clinical criteria are not relevant for the case definition since 2015. From 2001 to 2016, also cases fulfilling only laboratory criteria were transmitted to RKI.

#### Case definition before 2015

Cases fulfilling the case definition category

‘Clinical and Laboratory’: clinically and laboratory-confirmed hepatitis B cases.

Until 2015, the case definition required clinical confirmation of acute hepatitis and either direct (HBsAg or HBV-DNA) or indirect (IgM-antibodies to hepatitis B core antigen (anti-HBc-IgM)) detection of the virus (clinically and laboratory-confirmed cases) (see Table 1).

**Table 1.** Summary of hepatitis B case definitions in Germany before and since 2015

	Before 2015	Since 2015
Laboratory criteria	Direct or indirect detection of HBV: HBV-DNA confirmed HBs-Ag anti-HBc-IgM	Direct detection of HBV: HBV-DNA confirmed HBs-Ag
Clinical criteria	Clinical signs of acute hepatitis	Not relevant
Case definition	‘Clinical and Laboratory’: clinically and laboratory-confirmed hepatitis B cases	‘Clinical and Laboratory’: clinically and laboratory-confirmed hepatitis B cases ‘Laboratory without symptoms’: - laboratory-confirmed hepatitis B cases without clinical confirmation ‘Laboratory only’: laboratory-confirmed hepatitis B cases without information on clinical symptoms

#### Case definition since 2015

Cases fulfilling the case definition categories

(1) ‘Clinical and Laboratory’: clinically and laboratory-confirmed hepatitis B cases.
(2) ‘Laboratory without symptoms’: laboratory-confirmed hepatitis B cases without clinical hepatitis signs.
(3) ‘Laboratory only’: laboratory-confirmed hepatitis B cases without information on clinical symptoms.

Since 1 January 2015, only direct detection of HBV fulfils the laboratory criteria.

To apply the new case definition since 2015, a software update was necessary in the LPHA. Those still using old versions applied the old case definition. Case definitions are applied by the LPHA only. If cases transmitted in 2015 and 2016 were categorised according to the old case definition in place before 2015, the RKI did not re-apply the changed case definition.

#### Asylum seeker cases

Since September 2015, detailed information on the asylum seeker status has been collected in the surveillance system through a tick box or free text in the comment field. This additional information includes country of birth and date of arrival in Germany. Asylum seeker cases were defined as hepatitis B cases that were labelled by the LPHA as such in the notification data.

#### Cases with unknown asylum seeker status

For cases without information on the asylum seeker status it was impossible to discriminate if these were non-asylum seekers (residents) or if the information was missing. Therefore, cases without information were defined as cases with the unknown asylum seeker status. These could include either non-asylum seekers (residents) or misclassified asylum seekers.

#### Most probable country of infection

The information on the most probable country of infection was investigated by the LPHA and transmitted as additional information in the notification software if available.

#### Implications of the changed case definition

The positive-predictive value (PPV) of the case definition was defined by the proportion of transmitted hepatitis B cases that fulfil the case definition that was in place at the time when cases were reported on all transmitted hepatitis B cases [21]. The PPV was calculated per year of notification including cases according to the old and the changed case definition dependent on the software used in the notifying LPHA. The PPV was also calculated according to the case definition that was applied in the LPHA at the time the case was reported. We compared proportions of all transmitted cases by the case definition category in 2015 and 2016 to baseline (2010–2013).

#### Analysis of the hepatitis B cases identified through screening of asylum seekers

The district and according federal state of a case was defined by the LPHA that investigated and transmitted the case, in general this is concordant with the address of the patient's place of residence. Reporting incidence was calculated as the number of cases per 100 000 inhabitants. Descriptive analysis by the asylum seeker status, year of notification and district was performed. Federal states with obligatory screening for hepatitis B in asylum seekers were compared with federal states without regular screening. To determine if asylum seeker cases had acquired the infection in Germany or abroad we compared the most probable country of infection, as defined by the LPHA, and year of entry to Germany to the year of notification.

#### Analysis of the excess of hepatitis B cases with unknown asylum seeker status in the years 2015 and 2016

Cases with the unknown asylum seeker status were analysed by year of notification and compared to asylum seeker cases. As a first step, we fitted a Poisson regression to the number of hepatitis B cases between 2010 and 2013 stratified by the federal state, age group (<40 years/≥40 years) and sex. We also checked a negative binomial regression, but did not find a significantly improved fit to the data. We assumed a constant baseline over time, since in most federal states there was no significant secular trend, and in the few federal states with an increasing or decreasing trend, this trend seemed to be too instable to be extrapolated to future years. Then we simulated the 95%-prediction interval from the Poisson model. The number of excess cases was computed as the number of observed cases above the prediction interval, i.e. the positive part of the difference of the number of observed cases and the upper bound of the prediction interval. This excess number of cases was computed for the years 2015 and 2016 and – to double check the quality of the model fit – for the time period 2010 to 2013. The age group and sex distribution was then compared for excess cases and asylum seeker cases.

#### Implications of the changed case definition on the capture of asylum seeker cases

To identify asylum seeker cases only captured because of the changed case definition, we compared the proportion of asylum seeker cases and cases with the unknown asylum seeker status within case definition categories.

#### Statistics

To compare the proportions *χ*^2^ test was used.

The Wilcoxon–Mann–Whitney-test was used to compare medians.

## Results

The total number of transmitted cases to the RKI, irrespective if the case definition was fulfilled, increased from a mean of 1855 cases per year in 2010–2013 to 3873 cases in 2015 and 3466 in 2016. This represents an absolute increase of 2018 cases in 2015 and 1611 cases in 2016.

### Implications of the changed case definition

**Fig. 1.**
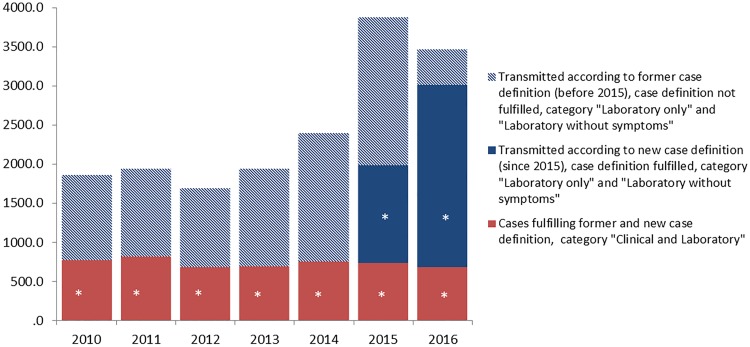
Number of transmitted hepatitis B cases according to case definition, case definition category and year of notification during the transition phase of implementation of the new case definition since 2015, Germany 2010–2016. * Cases published in official statistics.

The PPV of transmitted cases fulfilling the case definition applied by the LPHA increased substantially: in 2010–2013 40% of transmitted cases (*n* = 736) on average fulfilled the case definition which was applicable before 2015. In 2015, 51% (*n* = 1982) and in 2016, 87% (*n* = 3006) of transmitted cases fulfilled the case definition applied by the LPHA, either the case definition before or since 2015 (see Fig. 1). In 2015, the software update to transmit cases according to the changed case definition has not been implemented in all LPHA: 62% of cases (*n* = 2392) were transmitted according to the case definition which was applicable before 2015. This decreased in 2016 to 18% of cases (*n* = 629). The PPV of cases fulfilling the case definition which was in place before 2015 was 34%, of cases fulfilling the case definition since 2015 100% (*P*-value <0.01).

Cases transmitted in the categories that only fulfil the case definition since 2015 (‘Laboratory without symptoms’ and ‘Laboratory only’) increased significantly from a mean of 60% (*n* = 1119) in baseline (2010–2013) to 81% in 2015 (*n* = 3139) and 80% in 2016 (*n* = 2783) (*P* < 0.01). The number of cases with clinical information and transmitted within the categories ‘Clinical and Laboratory’ and ‘Laboratory without symptoms’ did not substantially increase since 2015. The observed increase of transmitted cases since 2015 is only attributable to cases within category ‘Laboratory only’ for which no clinical information is available at LPHA: from a mean of 252 cases in baseline (2010–2013) cases increased by 2296 in 2015 and by 1888 in 2016 (*P* < 0.01).

### Hepatitis B cases identified through screening of asylum seekers

**Fig. 2.**
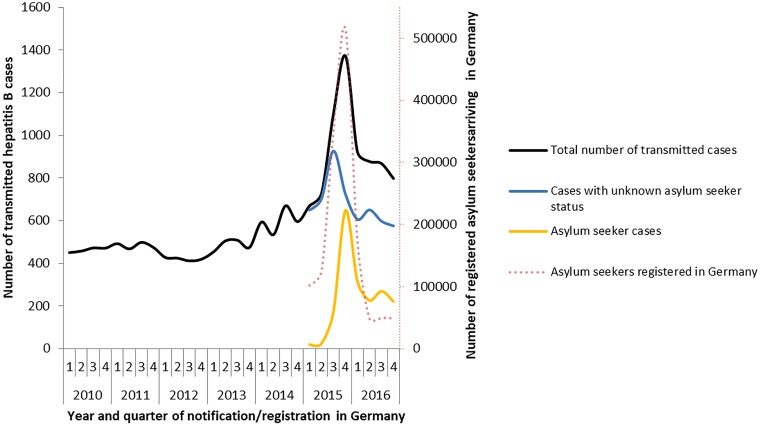
Number of transmitted hepatitis B cases in 2010–2016 according to the asylum seeker status and the number of registered asylum seekers in Germany in 2015–2016 per quarter (due to the time delay in processing applications for asylum, asylum seekers were registered on arrival in Germany [15, 16, 20]).

In 2015, 869 (22%) of all transmitted hepatitis B cases were known asylum seekers. This increased in 2016 to 1034 (30%) cases. The increase of transmitted cases started in 2015 before information on asylum seekers was available in the surveillance system (second half of 2015, see Fig. 2). The increase of cases in the surveillance system was observed in the same time period as the increase in registered asylum seekers in Germany.

Reporting incidence of hepatitis B cases varied in different federal states over time (see Table 2). In few federal states case numbers remained unaffected in 2015 and 2016 compared to baseline (e.g. Thuringia), whereas in most federal states case numbers increased in 2015 compared to baseline (e.g. Hamburg, Bavaria). Only in one federal state, the reporting incidence declined in both, 2015 and 2016, compared to baseline (Bremen).

**Table 2.** Number of transmitted hepatitis B cases/100▫000 inhabitants for each federal state in 2010–2013, 2015 and 2016 according to the asylum seeker status, and difference in reporting incidence (total) Germany

	Hepatitis B reporting incidence					Difference to baseline	
Federal state	Mean 2010–2013 (baseline)	2015		2016		2015	2016
	Total	Total	Asylum seeker cases (%)	Total	Asylum seeker cases (%)	Total	Total
Hamburg ^a^	2.9	36.3	64	8.9	61	+33.4	+6.0
Bavaria ^a^	2.6	8.2	27	7.2	48	+5.6	+4.6
Saxony ^a^	3.7	6.5	14	8.0	35	+2.7	+4.3
Hesse	3.3	6.1	7	6.3	16	+2.8	+3.0
Schleswig-Holstein	1.9	4.5	1	3.7	8	+2.6	+1.8
Mecklenburg-Western Pomerania	0.9	2.2	5	2.9	14	+1.3	+2.0
Baden-Wurttemberg	1.9	3.0	5	3.5	23	+1.1	+1.6
Saxony-Anhalt	2.0	3.3	21	3.2	48	+1.4	+1.2
Brandenburg	1.7	2.4	15	3.2	30	+0.8	+1.6
Rhineland-Palatinate	2.9	2.4	11	4.7	20	−0.6	+1.8
Lower Saxony	1.1	1.7	36	1.7	51	+0.6	+0.6
North Rhine-Westphalia	1.5	2.1	5	2.0	10	+0.6	+0.4
Berlin	6.6	7.0	6	6.7	15	+0.3	+0.1
Thuringia	0.7	0.9	5	0.7	38	+0.2	+0.0
Saarland	3.2	3.3	0	2.1	0	+0.1	−1.1
Bremen	2.4	0.7	0	1.3	22	−1.6	−1.1
Total	2.3	4.7	22	4.2	30	+2.5	+2.0

a Federal states that conducted hepatitis B screening in asylum seekers in 2015 [17–19, 22].

According to information on the surveillance data, 98% of asylum seeker cases in 2015 and 66% in 2016 had entered Germany in the same year their hepatitis B infection was notified. Of 422 asylum seeker cases (22%) in 2015–2016 with information on probable country of infection, in 50 cases (12%) Germany was reported as probable country of infection.

### Excess of hepatitis B cases with unknown asylum seeker status in the years 2015 and 2016

In 2015 and 2016, asylum seeker cases were younger than cases with the unknown asylum seeker status (median age 25 *vs.* 37 years, *P* < 0.01) and more likely to be male (80% *vs.* 63%, *P* < 0.01). Cases with the unknown asylum seeker status differed in 2015 and 2016 to cases in 2010–2013 (baseline): In 2015, cases with the unknown asylum seeker status were younger than those in 2010–2013 (median age 36 *vs.* 41 years, *P* < 0.01) and more often male (65% *vs.* 62%, *P* < 0.01). In 2016, cases with the unknown asylum seeker status were younger than in baseline (median age 39 *vs.* 41 years, *P* < 0.01).

**Fig. 3.**
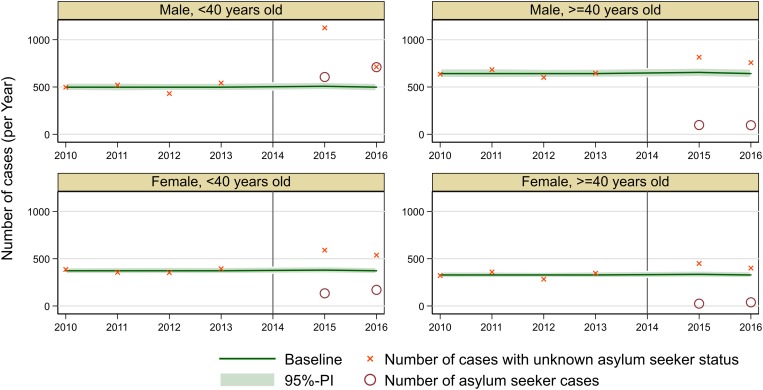
Summary of expected number of cases in 2015–2016 with 95% prediction interval (PI) and real observed number of cases according to the asylum seeker status, Germany 2010–2016.

The excess of cases with the unknown asylum seeker status was calculated to be 766 cases in 2015 and 333 cases in 2016 (see Fig. 3). Overall, the excess of cases with the unknown asylum seeker status consisted mainly of male cases younger than 40 years. Whereas in 2010–2013, 20% of cases were males younger than 40 years, this was higher in asylum seeker cases (69%, *P* < 0.01). The excess of cases with the unknown asylum seeker status was similar to asylum seeker cases in 2015 (68% males <40 years, *n* = 520 cases, *P* = 0.4), but in 2016 the excess of cases with the unknown asylum seeker status was different from asylum seeker cases (45% males <40 years, *n* = 151 cases, *P* < 0.01) (see Table 3).

**Table 3.** Comparison of demographics of excess cases with the unknown asylum seeker status, Germany 2010-2016.

Sex	Age group (in years)	2010–2013 *n* (%)	2015 *n* (%)	2016 *n* (%)
Male	Total	14 (41%)	596 (78%)	208 (62%)
	<40	11 (32%)	520 (68%)	151 (45%)
	≥40	3 (9%)	76 (10%)	57 (17%)
Female	Total	20 (59%)	170 (22%)	125 (38%)
	<40	8 (24%)	123 (16%)	89 (27%)
	≥40	12 (35%)	47 (6%)	36 (11%)
Total	Total	34 (100%)	766 (100%)	333 (100%)

### Implications of the changed case definition on the capture of asylum seeker cases

Asylum seeker cases in 2015 and 2016 were mostly transmitted according to the changed case definition: 1759 asylum seeker cases (92%) were transmitted within the case definition categories ‘Laboratory without symptoms’ and ‘Laboratory only’, compared to 4163 cases with the unknown asylum seeker status (77%). Asylum seeker cases were significantly more likely to be transmitted within case definition categories without clinical information (*P* < 0.01).

## Discussion

The increase of hepatitis B cases published in official statistics in Germany has been partly caused by the change in case definition in 2015. The change in the case definition was important to include all active hepatitis B cases in the surveillance system, regardless of the information on clinical symptoms. Cases without information on clinical symptoms were already transmitted to the national level before 2015, but were not published as they did not fulfil the case definition by then. Therefore, the more sensitive case definition since 2015 led to an expected increase in the number of published cases, but did not affect the overall number of transmitted cases. Classification of cases according to the case definition depends on the software used. A software update, which was necessary to transmit cases according to the changed case definition, has been implemented stepwise in the LPHA. During 2015 and 2016, at the same time some LPHA applied the old and others the changed case definition. As the PPV of the changed case definition since 2015 was significantly higher and the proportion of LPHA applying the old case definition decreased over time, this lead to an increase in published case numbers. Therefore, the time of implementation in each LPHA influenced the number of transmitted cases fulfilling the case definition and had an impact on the number of published cases. We believe that the changed laboratory criteria in 2015 had only a minor effect on the number of notified cases, but the actual impact of this remains unknown and should be investigated further. Nevertheless, the number of transmitted cases without clinical information has increased since the change in case definition in 2015 leading to poorer data quality.

A substantial increase in the number of transmitted cases was due to the large influx of asylum seekers originating mostly from high prevalence countries for hepatitis B into Germany, and hepatitis B screening activities within this population. A study conducted in a reception centre for immigrants in Northern Germany found a prevalence of active hepatitis B of 2.3% [23]. This is in accordance with screening results obtained in Bavaria, where 3.3% of asylum seekers were diagnosed with active hepatitis B [18]. The increase in the number of cases varied between federal states and districts. Regulations on screening practices for asylum seekers alone cannot explain the increase in all federal states as also federal states without such a regulation observed an increase in reporting incidence of hepatitis B. In federal states with the highest increase in reporting incidence (Hamburg, Bavaria and Saxony), hepatitis B screening was conducted for all asylum seekers. How screening regulations have been put into practice on the district level and if federal states without such a regulation performed a systematic screening for hepatitis B in asylum seekers remains unclear. A study conducted from June to October 2015 in four federal states found that 14 of 33 surveyed local public health authorities screened all asylum seekers for hepatitis B, whereas 13 screened only a subset (including symptomatic individuals) and 6 did not perform a screening [22]. In 2015, the incidence of chronic hepatitis B cases has risen in European countries according to ECDC due to increased local testing among migrants and other key populations [4] as migrants in European countries account for 25% of all chronic hepatitis B cases [24]. This is also supported by the finding that the highest increase of hepatitis B reporting incidence in 2015 compared to 2010–2013 was found mostly in countries that also recorded the highest number of registered asylum seekers compared with the population in this year (such as Sweden, Austria, Finland and Germany) [25]. The incidence of hepatitis B should be interpreted carefully because it is an under-diagnosed chronic disease. Epidemiology of other infectious diseases was impacted by the influx and screening of asylum seekers, too [26]. Referral of asylum seekers from one district or federal state to another might have led to double registration and potentially double screening for hepatitis B and, therefore, might have increased the number of transmitted cases. It might also have led to incorrect allocation of cases to federal states.

The majority of hepatitis B infections in asylum seeker cases was imported from abroad and not acquired in Germany, which is not surprising considering that most of them came from countries with high prevalence [12–14]. However, interpretation of this information is difficult because of poor data quality as the information on the probable country of infection was only available for a subset of cases and as this information was determined by the LPHA without further information on the criteria applied. Interpretation of this information is especially aggravated in chronic infectious diseases. In other European countries hepatitis B infection was imported for 61% of reported cases in 2015 [4].

Screening of asylum seekers might have influenced the number of hepatitis B cases even to a higher extent than the notification data indicates. Changes in age and sex distribution amongst cases with the unknown asylum seeker status before and after 2014 suggest that, in reality, many asylum seekers might be amongst the cases with the unknown asylum seeker status, especially in 2015. This is supported by our results on the excess of cases with the unknown asylum seeker status, although our model might even underestimate the excess. Collection of information on the asylum seeker status has been implemented in the surveillance system in late 2015 and the necessary software update has been implemented in LPHA only stepwise afterwards. In addition, difficulties in case investigation in asylum seeker cases due to language barriers or relocation might have contributed to more unknown information (e.g. clinical information, probable country of infection) in these cases. This could explain why asylum seeker cases are more often transmitted without clinical information (category ‘Laboratory only’). Without the change in case definition all these cases would not have been published as they did not fulfil the case definition before 2015. Information on true numbers and demographics of asylum seekers coming to Germany is scarce and comes with a large time delay, especially in 2015.

According to the Protection against Infection Act only acute hepatitis B infections were notifiable to LPHA until 2017. We believe that laboratories notified all newly diagnosed hepatitis B cases, irrespective of their infection stage, even before the adaption of the law as it is difficult to discriminate infection stages on the basis of laboratory findings only and the notification data contains also cases with unknown stage and known chronic infections. Since 2017 all active hepatitis B cases are notifiable in Germany. This revised Act also emphasised the need to improve the quality of surveillance data regarding country of birth and whether an infection is considered to be imported as ECDC recommends [4]. Based on our analysis we revised analysis, display and publishing of hepatitis B surveillance data, e.g. within the annual report on infectious diseases [27].

## Conclusion

The change in case definition increased the number of hepatitis B cases published in official statistics substantially. Influx and screening of asylum seekers increased the number of transmitted cases even to a higher extent than the surveillance data indicates. The epidemiology of hepatitis B in Germany might have changed due to the influx of asylum seekers from countries with high prevalence. The change in case definition was essential to publish all cases with active hepatitis B including cases with limited or missing clinical information. Especially, asylum seeker cases would not have been captured in published data without the change in case definition.

## Recommendation

Information on the asylum seeker status is valuable and completeness should be improved. One step towards this has been taken by adding country of birth and year of entry to Germany as mandatory information in the revised Protection against Infection Act in 2017. To better understand the impact of the influx of asylum seekers on the epidemiology of infectious diseases in Germany, surveillance data should be followed up in the next years and reliable information on screening practices within federal states and districts should be made available. To support the health of asylum seekers and prevent the increased spread of hepatitis B in Germany health assessment immediately after arrival, universal vaccination of infants and children against hepatitis B and vaccination based on other indications according to the Standing Committee on Vaccination recommendations should be ensured.
